# Increasing bird diversity in a landscape in transition

**DOI:** 10.1007/s00442-026-05948-9

**Published:** 2026-09-11

**Authors:** António E. Monteiro, António Vaz Pato, Luís Reino, Fernando Silla, Henrique M. Pereira

**Affiliations:** 1 Departamento de Biología Animal, Ecología, Parasitología, Edafología y Química Agrícola, Universidad de Salamanca, Salamanca, Spain; 2 BIOPOLIS Program in Genomics, Biodiversity and Land Planning, CIBIO, Campus de Vairão, Vairão, 4485-661 Portugal; 3 Faculdade de Ciências da Universidade de Lisboa, Campo Grande, Lisboa, 1749- 016 Portugal; 4 German Centre for Integrative Biodiversity Research (iDiv) Halle–Jena– Leipzig, 04103 Leipzig, Germany; 5 Institute of Biology, Martin Luther University Halle-Wittenberg, 06108 Halle (Saale), Germany

**Keywords:** Biodiversity change, Land abandonment, Rewilding, Mediterranean uplands, Species richness, Community assemblage, Monitoring

## Abstract

Rural depopulation across Mediterranean uplands has driven farmland extensification and landscape transitions, yet their medium-term impacts on birds remain poorly understood. We resurveyed 213 selected 2 km × 2 km grid cells in the Côa River basin (western Iberia) in 2023–2024, following protocols comparable to those applied in 1999–2005, ensuring consistent sampling across periods. Median alpha diversity increased by five species, a 28% gain, with total abundance rising as common taxa became more widespread. Gains were predominantly driven by forest-associated and generalist species, while farmland specialists showed stagnation or slight declines. We discuss how landscape transitions contributed to these biodiversity patterns including lower human density, reduced grazing pressure, and altered disturbance regimes. However, significant avian diversity gains occurred within apparently stable forest-cover mosaics, potentially masking broader ecological shifts.

**Supplementary Information:**

The online version contains supplementary material available at https://doi.org/10.1007/s00442-026-05948-9.

## Introduction

Rural depopulation is an increasingly pressing issue in Europe, particularly in the context of globalization and the uncertain socio-ecological future of sparsely populated regions. It is also a major driver of farmland abandonment, leading to landscape transitions that pose both challenges and opportunities for biodiversity and ecosystem services (Queiroz et al. 2014; Navarro and Pereira 2012; Fernández et al. 2020). In this context, regions of the Mediterranean biome, a globally recognized biodiversity hotspot facing escalating climatic change (Blondel and Aronson 1999), emerge as priority areas for investigating ongoing ecological shifts (Queiroz et al. 2014; García-Ruiz et al. 2020).

Northeastern Portugal has a relatively sparse human population (in Western Europe context) and has been undergoing rural depopulation. Since the mid-twentieth century, its population has declined by over 70%, cereal cropping has fallen by 90%, and traditional pastoralism has almost disappeared (INE 2021; Sa-Rego et al. 2022; Eurostat 2025). These landscapes hold high biodiversity value, particularly for birds, and since the 1990s large portions have been designated as protected areas under Natura 2000, including three distinct Special Protection Areas (SPAs) under the Birds Directive (ICNF 2024).

The analysis of avian community dynamics offers a robust empirical framework for elucidating the interplay between biodiversity and landscape changes. There are multiple decades-long bird monitoring initiatives across Europe, including through national and regional atlas programs, which have yielded some of the most robust time series available for any vertebrate group (Brlík et al. 2021; PECBMS 2025). In many regions, declining human activity and land abandonment have driven structural changes in the landscape, with uneven effects on avian communities (Preiss et al. 1997; Salaverri et al. 2019). Long-term monitoring datasets have demonstrated that species gains or losses are contingent upon their ecological traits, habitat preferences, and functional specificity (Burns et al. 2021; Gregory et al. 2023). Some studies, particularly in Mediterranean and temperate landscapes undergoing farmland abandonment, have documented declines not only in species richness but also in functional diversity, indicating a loss in the range of ecological roles that birds perform within communities. For instance, Salaverri et al. (2019) reported functional erosion in Spanish cereal-steppe bird assemblages over two decades, associated with shrub encroachment and loss of open habitats. Oksuz et al. (2021), working in Anatolian grasslands, found that woody vegetation expansion reduced the abundance of steppe specialists and narrowed trait diversity. Similarly, Losada et al. (2023) observed in north-western Iberia that succession toward forest cover after agricultural abandonment contributed to the decline of grassland and shrubland birds. Conversely, other studies highlight that the observed increase in tree and shrub cover, along with improved structural habitat connectivity, has benefited a broad range of species. For example, Gil-Tena et al. (2007) documented positive responses of forest specialists to increased landscape connectivity in Catalonia, while Guilherme and Pereira (2013) found that passive rewilding in the Peneda-Gerês favoured the recolonization by forest birds. Edo et al. (2023) also reported higher alpha and beta diversity in Mediterranean agroforestry systems compared to open farmland, driven partly by gains in forest-associated species.

At the same time, broad-scale monitoring schemes have identified some increases in abundance across certain species and regions (Burns et al. 2021; PECBMS 2025), including large-bodied soaring birds such as vultures, which have shown consistent recoveries in Europe over recent decades due to reduced human pressures and expanding natural cover (Cortés-Avizanda et al. 2015; Martínez-Abraín et al. 2020). Few studies analyse long-term changes in species richness, functional reassembly, or structural shifts in Mediterranean low-density interior regions (Preiss et al. 1997; Brotons et al. 2018; Brlík et al. 2021). Covering over 50% of southern Europe (Eurostat 2024), these depopulated landscapes remain ecologically understudied, with the pace and direction of transitions in ecosystem services still uncertain (Fusco et al. 2021; Bruno et al. 2021).

In this study, we investigate the evolution of species richness and the dynamics of functional and habitat groups in bird communities at the regional landscape in Mediterranean uplands in Northeast Portugal. The main goals of this study are to (i) quantify changes in local species richness between 1999 and 2005 and 2023–2024, (ii) analyse the structural and functional reconfiguration of bird assemblages, and (iii) inform conservation and monitoring strategies for similarly low-intensity landscapes across the Mediterranean.

We hypothesise that bird communities have undergone measurable shifts in species composition, relative dominance, and ecological function, reflecting landscape transition associated with human presence decline. Finally, we discuss the implications of these transformations for rewilding, biodiversity monitoring, and ecological resilience under ongoing socio-economic change.

## Methodology

### Study area

**Fig. 1:**
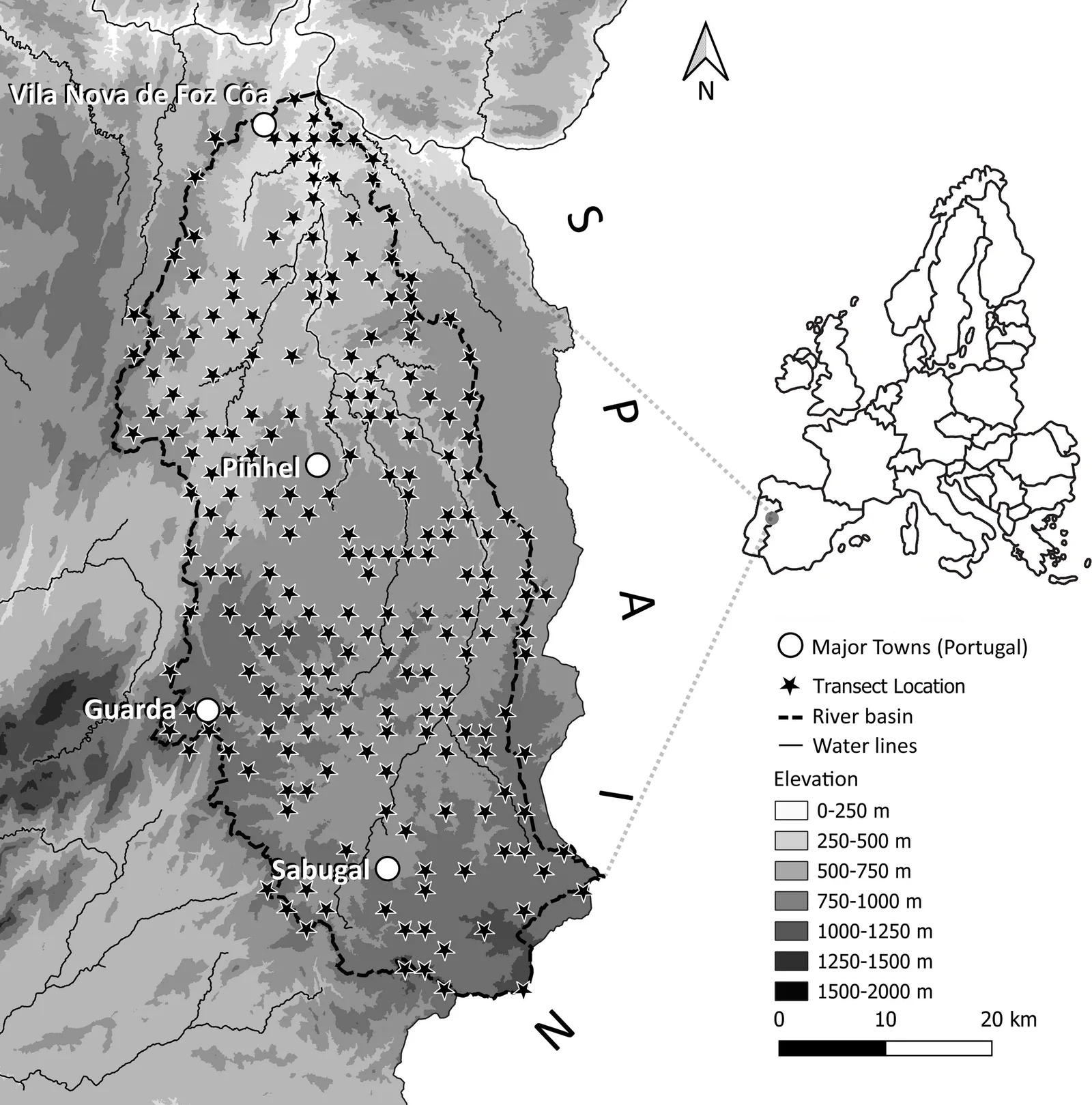
The study area, Côa River Basin (Portugal), located in the central region of Portugal within the Iberian Peninsula. Stars represent the 213 sampled squares

We conducted the study in the 2,521 km² Côa River Basin in northeastern Portugal, a broad granite-schist plateau dissected from north to south by the Côa River and its tributaries (Jorge 2009; Fig. 1). Two Mediterranean bioclimatic belts influence the region. Above approximately 500 m, a Mesomediterranean zone features dry summer climates (Csa) with > 600 mm of annual rainfall, while lower areas fall into Thermomediterranean zones, which receive < 300 mm annually and experience hotter, drier conditions (Mora and Vieira 2020; AEMET and IM 2011; Monteiro-Henriques et al. 2016). Vegetation reflects this gradient, ranging from xerophytic *Cytisus-Lavandula* shrublands and terraced orchards in the lowlands to mixed oak woodlands (*Quercus ilex*,* Q. suber*,* Q. pyrenaica*) and remnant *Pinus pinaster* plantations at higher elevations (Monteiro-Henriques 2021).

## Land use change

Since the 1950s, the region experienced a steep decline in human population, with an average decrease of nearly 10% per decade in the 21st century (INE 2023). In recent decades, population densities fell below 20 inhabitants km², with many areas averaging fewer than 10 km² (INE 2023; Viana et al. 2021; Eurostat 2025). This demographic collapse led to extensive land-use change (Fig. S1 - Online Resource 1), marked by a > 90% decline in cereal cultivation and sharp reductions in sheep grazing (Honrado et al. 2017; INE 2021; Sa-Rego et al. 2022). Traditional agro-pastoral systems largely disappeared, though some subsistence plots and orchards persisted near villages (Honrado et al. 2017; Pereira et al. 2022). This shift triggered widespread shrub encroachment, natural forest recovery in marginal areas, and reestablishment of riparian vegetation (Elphick et al. 2024). More recently, extensive cattle grazing became widespread, accompanied by a reduction in wildfire frequency (INE 2021; Kirkland et al. 2024; AGIF 2025; EFFIS 2025).

To determine the changes in land use in the last 30 years, we used the Land Use Charts from 1995 to 2018 as the reference years closest to our sampling period. We considered only three major land use categories for our analysis: (1) Farmland (annual and permanent crops, pastures); (2) Shrubland (shrubs and scrub areas); and (3) Forest (production forest, semi-natural woods and agroforestry systems). Using QGIS software (QGIS 2024), we juxtaposed both charts to identify transfers of land use (in square kilometers) between these three categories, which we summed up in a Sankey Diagram (Fig. S2 - Online Resource 2).

Current land use data from 2018 (DGT 2020) showed agriculture still occupied approximately 40% of the basin, primarily extensive pastures and permanent crops like olives, almonds, and vineyards (all three concentrated in the north). Forest and shrublands each represented more than 30%, with forest expansion most pronounced in the southern uplands (DGT 2020; INE 2021). The resulting landscape formed a dynamic mosaic of cropland, pasture, shrubland, and forest at varying recovery stages. About 20% of the basin falls within Natura 2000 and national protected areas, including Serra da Malcata, the Côa Valley, and Douro Internacional (ICNF 2024).

## Study design and bird data

Changes in bird species richness over time were assessed by replicating, in 2023–2024 (hereafter T2), the standardized surveys originally carried out between 1999 and 2005 (hereafter T1) as part of the 2nd Portuguese Breeding Bird Atlas (Equipa Atlas 2008). The analysis focused on 213 4 km² sampling squares across the Côa River basin, covering approximately 250,000 ha (Fig. 1), a considerably large sample size which allows robust comparisons between periods. These squares corresponded to those surveyed during the original atlas period (T1) and were revisited in 2023–2024 to maximize comparability between periods. No formal procedure was applied to minimize spatial autocorrelation, and some neighbouring squares were contiguous.

Field surveys followed an unlimited-distance transect approach, whereby all birds detected within each 2 × 2 km sampling square during a fixed 30-minute period were recorded (Bibby et al. 2000). Fieldwork was preferentially conducted under suitable weather conditions and during periods of higher bird activity, particularly in the morning. Detections were based on both visual and auditory observations, and binoculars were used to assist species identification when necessary. Transect routes were not fixed to a predefined line but were selected within each square to maximize habitat coverage, following standard atlas protocols. Transects followed standardized protocols consistent with those applied in the Portuguese breeding bird atlas (1999–2005), which in turn were based on the methodology of the UK breeding bird atlas (Gibbons et al. 1993; Equipa Atlas 2008). To minimize the influence of adverse weather on species detectability, transects yielding fewer than 15 species were excluded, as such low counts were considered more likely to reflect meteorological constraints or reduced detectability rather than actual community composition (Lõhmus and Kaasik 2025).

In T1 surveys, sampling effort was standardized by survey duration and spatial unit rather than by distance travelled. Observers were instructed to walk at a regular and constant pace during 30-minute surveys, typically covering approximately 1 km per transect. These procedures helped ensure methodological consistency between periods and reduced variability associated with walking speed, time of day, and observer behaviour. T1 surveys involved 13 experienced observers participating in the second Portuguese Breeding Bird Atlas. The mean number of species detected per transect across the 13 main observers in T1 showed low variability and sufficient consistency to eliminate possible observer bias (mean = 21.17 ± 2.06 SD; range = 17.57–24.45).

In 2023–2024 (T2), all 213 sample squares were revisited by one observer and a new transect was carried out in each square following the same methodological criteria described above. Due to the lack of precise GPS records from the original surveys, the new transects were conducted within the same square boundaries, although the exact previous routes could not be replicated. This approach prioritized consistency in sampling units between periods, even if exact route replication and full spatial independence among sample squares could not be ensured. Occurrence data used in this study were submitted to the open-access *eBird.org* platform (Sullivan et al. 2009), which enabled standardized extraction with complete metadata for subsequent analysis. The full baseline occurrence matrices for T1 and T2 are provided in Table S1 and Table S2 (Online Resource 3). This study involved no capture or handling of animals and required no permits from the Portuguese Forestry and Conservation Institute (Instituto da Conservação da Natureza e das Florestas, ICNF).

## Bird diversity metrics

Bird community metrics were assessed using species alpha richness, defined as the number of species detected per sample square, which served as the main response variable (Whittaker 1972; González-Taboada et al. 2007). Species richness was quantified across 59 functional groups defined by ecological attributes, including habitat association, diet (granivores, insectivores, carnivores, frugivores, omnivores), foraging strata (ground and canopy foragers), migratory status, body mass, edge specialization and taxonomic affiliation. Habitat guilds were classified into four groups: farmland, forest, shrubland and generalist species, based on established ecological criteria for European birds (Storchová and Hořák 2018). Diet composition, trophic guild, foraging strata, activity time, migratory strategy and body mass were compiled from global and European trait databases, including EltonTraits (Wilman et al. 2014), Storchová and Hořák (2018; Pigot et al. (2020). Species-specific trait values used to assign functional groups are provided in Table_S3 (Online Resource 4). To simplify the analysis, only habitat guilds were considered due to their explicit relation with landscape changes.

In addition to functional richness, changes in the relative abundance of species were evaluated. Since individual abundance data were unavailable, frequency of occurrence—defined as the number of squares in which a species was detected—was used as a proxy for relative abundance (Preston 1948; Brown et al. 1995). This approach allowed the detection of abundance turnover, such as the emergence of previously rare species or the decline of once-dominant taxa, within a consistent analytical framework. Species were ranked by frequency in T1 and grouped into quartiles representing occurrence classes from Q1 (rare species) to Q4 (widespread, dominant species). Quartile assignment was also treated as an abundance class, and the number of species belonging to each class was counted across all sampling squares. These thresholds were fixed in T1 and applied to T2 to assess net change within each occurrence class. Species-level changes in occurrence between T1 and T2, including habitat group and T1 quartile, are provided in Table S4 (Online Resource 5).

### Statistical analysis

All analyses were performed in R using RStudio (R Core Team 2024). To verify whether there were major differences in the number of species detected between both periods, we conducted rarefaction and extrapolation analyses based on incidence data using Hill numbers with the iNEXT package (Chao and Jost 2012). We calculated diversity estimates in T1 and T2 for q = 0 (species richness), q = 1 (Shannon diversity) and q = 2 (Simpson diversity), with extrapolations extended to 250 sampling units. Confidence intervals at 95% were used to assess differences between periods, with overlapping confidence intervals interpreted as indicating no significant differences.

Temporal variation in species richness was assessed using sea stack plots (package seastackplot; Stuart et al. 2024), which depict mean values, 95% confidence intervals and species-richness histograms. Total and functional group net change (Δ = T2 − T1), as well as quartile-specific species richness, were evaluated using boxplots based on the difference between T1 and T2, computed separately for each sampling unit and for each functional group. All tests were Wilcoxon signed-rank tests assessing whether the median change differed from zero (Fig. 3a and b).

To further complement the boxplot analysis on species relative abundance, we performed a Generalized Linear Model (GLM) with quasibinomial distribution due to residuals’ overdispersion. We plotted species relative abundance (as proportion of sites where the species was observed) in T1 as predictor and the relative abundance in T2 as response.

Net changes in species frequency of occurrence were examined by plotting boxplots using species as the unit while retaining the quartile classification described in Bird Diversity Metrics to evaluate shifts from rarer (Q1) to more frequent species (Q4). To further assess changes in species frequency of occurrence, Species Abundance Distribution (SAD) models were fitted using a log-Poisson distribution through the sads package (Prado et al. 2016). Two log₂-transformed frequency-of-occurrence histograms for T1 and T2 were overlaid with their respective model curves to visualize changes in community structure over time. In this type of model, a shift of the SAD maximum to the left or to the right between periods indicated either a decrease or an increase in the frequencies of occurrence of the species in the community.

## Results

### Changes in species richness over time

Between 1999 and 2005, we recorded a total of 117 bird species (112 breeding and 5 non-breeding), while in the second period (2023–2024), we detected 115 species (111 breeding and 4 non-breeding) Rarefaction and extrapolation analyses based on Hill numbers (Fig. S3 - Online Resource 6) showed very similar sample coverage – i.e. proportion of the total assemblage represented by the observed sample - between periods (T1: 0.997 T2: 0.998).

**Fig. 2:**
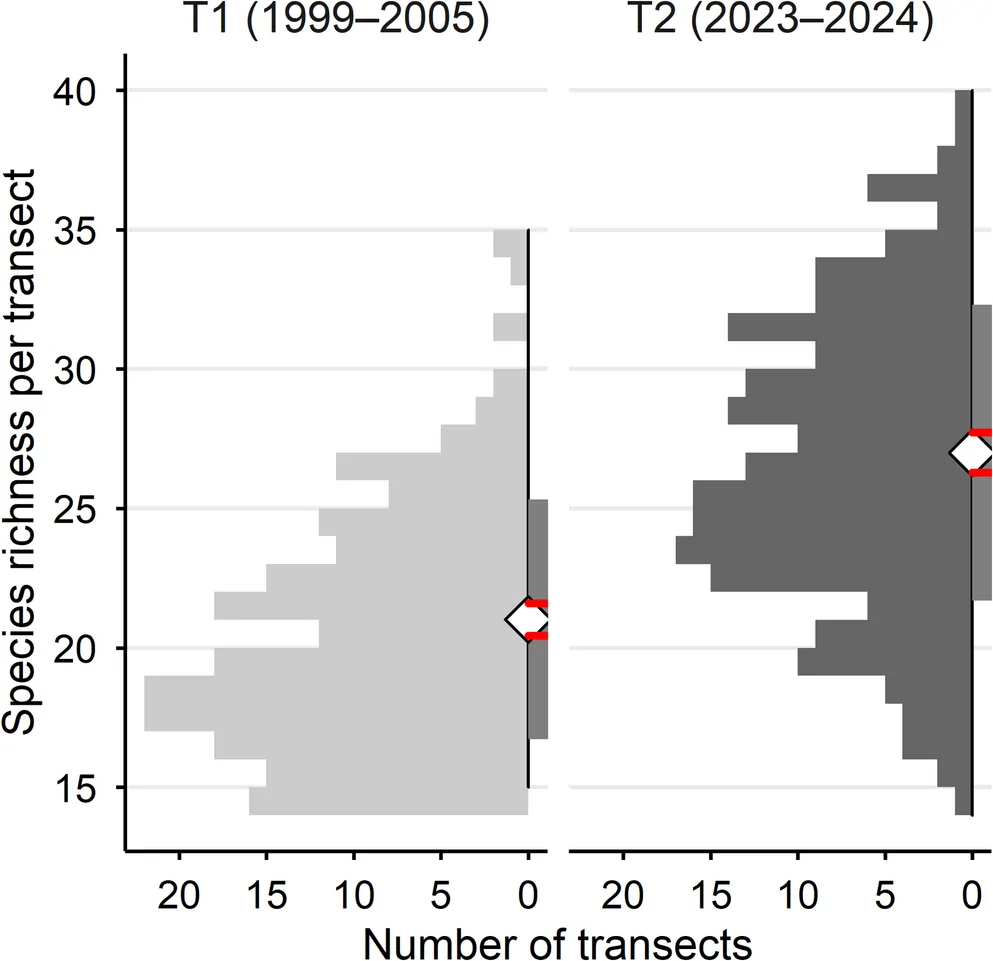
Sea stack plots depicting the variability of species richness in transects in T1 (1999–2005) and T2 (2023–2024). Each histogram bar represents the number of transects for each species richness value. Histograms show ± 1 standard deviation (grey vertical bar) from the mean (white diamonds) and 95% confidence intervals for the mean (red horizontal lines) (Color figure online)

Across both periods combined, a total of 131 species were recorded: 79 increased their frequency of occurrence between the two time periods, 48 decreased, and four remained unchanged. A species-level summary of occurrence frequencies and their temporal changes is provided in Table S4 (Online Resource 5). Sixteen species appeared exclusively in the first period, while fourteen were observed only in the second. Alpha diversity (mean species richness per transect) increased from 21.02 ± 4.29 (T1) to 27.00 ± 5.31 (T2) across the 213 sampling sites (Wilcoxon test, W = 8677.5, *p* < 0.001; Fig. 2). Of the 131 species, 36% were strictly associated with forest habitats (*n* = 47), 28% were generalists (*n* = 37), 15% were associated with shrubland (*n* = 19), 5% with farmland (*n* = 6), and 17% belonged to other categories (aquatic, rocky, mainly occasional species that not reflect the local breeding assemblage) (*n* = 22).

**Fig. 3:**
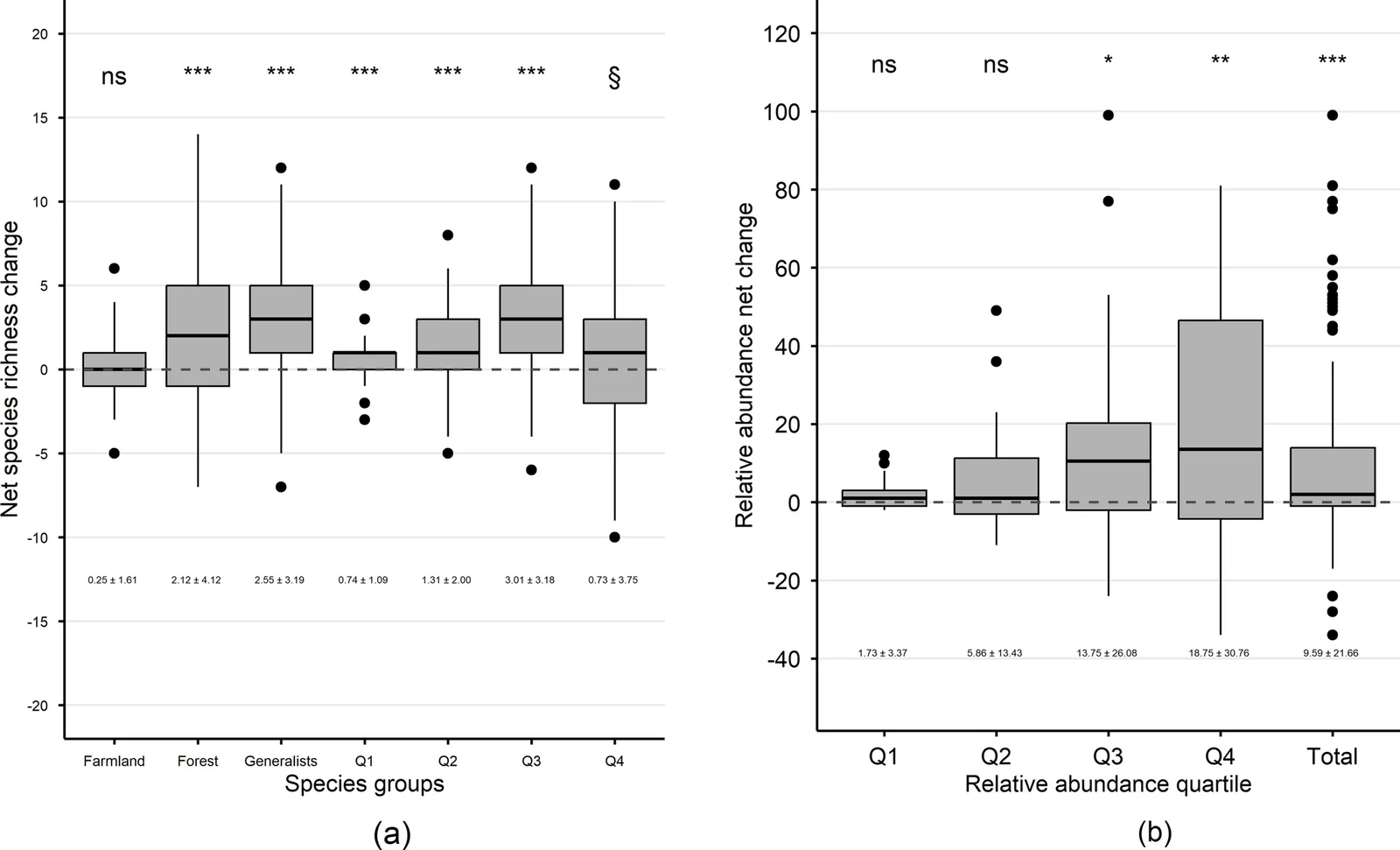
**a** Boxplots depicting net change in species richness for different species groups between the two time periods. First three boxplots from left to right refer to the three main habitat-use functional groups (farmland, forest and generalists), while the remaining four boxplots represent net changes in species for relative abundance quartiles in T1 (Q1 - rarer species in T1 sample - to Q4 - more frequent species in T1 sample). Positive values indicate higher values in T2, and negative values indicate higher values in T1. Quartile breaks were based on species frequency of occurrence in T1 (Q1 = rarest species; Q4 = most frequent species). The central horizontal line indicates the median, boxes represent the interquartile range, whiskers extend to 1.5 × the interquartile range, and black dots indicate outliers. A dashed horizontal line at y = 0 indicates no net change. Mean, standard deviation and p-values are shown below each boxplot. Significance levels for non-zero changes between the two time periods: *** *p* < 0.001, ** *p* < 0.01, * *p* < 0.05, § *p* < 0.1, ns *p* > 0.1. **b** Boxplots depicting net change in species relative abundance for, from left to right, the four species abundance quartiles and the overall species community. Positive values indicate higher values in T2, and negative values indicate higher values in T1. Quartile breaks were based on species relative abundance in T1 (Q1 - rarer species in T1 sample - to Q4 - more frequent species in T1 sample). The central horizontal line indicates the median, boxes represent the interquartile range, whiskers extend to 1.5 × the interquartile range, and black dots indicate outliers. A dashed line was drawn at y = 0 to facilitate visualization. Mean, standard deviation and p-values were added at the bottom of each boxplot. Significance levels for non-zero changes between the two time periods: *** *p* < 0.001, ** *p* < 0.01, * *p* < 0.05, § *p* < 0.1, ns *p* > 0.1

Changes in alpha diversity over approximately 20 years did not occur uniformly across groups. Species richness increased steadily among forest-associated and generalist species, while farmland-associated species showed no significant changes and remained broadly stable, albeit with slight declines in median richness (Fig. 3a). Forest-related guilds experienced mostly positive changes, farmland taxa showed low variation and no consistent directional change. Generalist species showed the strongest gains in alpha diversity, with a median increase of approximately three species per sampling square, equivalent to a mean rise of around 18% in site-level richness. Forest species followed a similar variation, with average increases of two species per site (a 14–16% gain). In contrast, farmland specialists exhibited little to no net change: site-level variation remained within ± 1 species, and mean changes stayed below 5%. Farmland species overall showed near-zero net change, with interquartile ranges capturing both slight gains and losses. On the other hand, quartile-based T1 frequency groups (Fig. 3a, Q1, Q2, Q3 and Q4 boxplots), showed average species richness gains across all quartiles, with more expressive gains amongst moderately frequent species groups (Q2 and Q3).

## Changes in relative abundances (species frequency of occurrence)

**Fig. 4:**
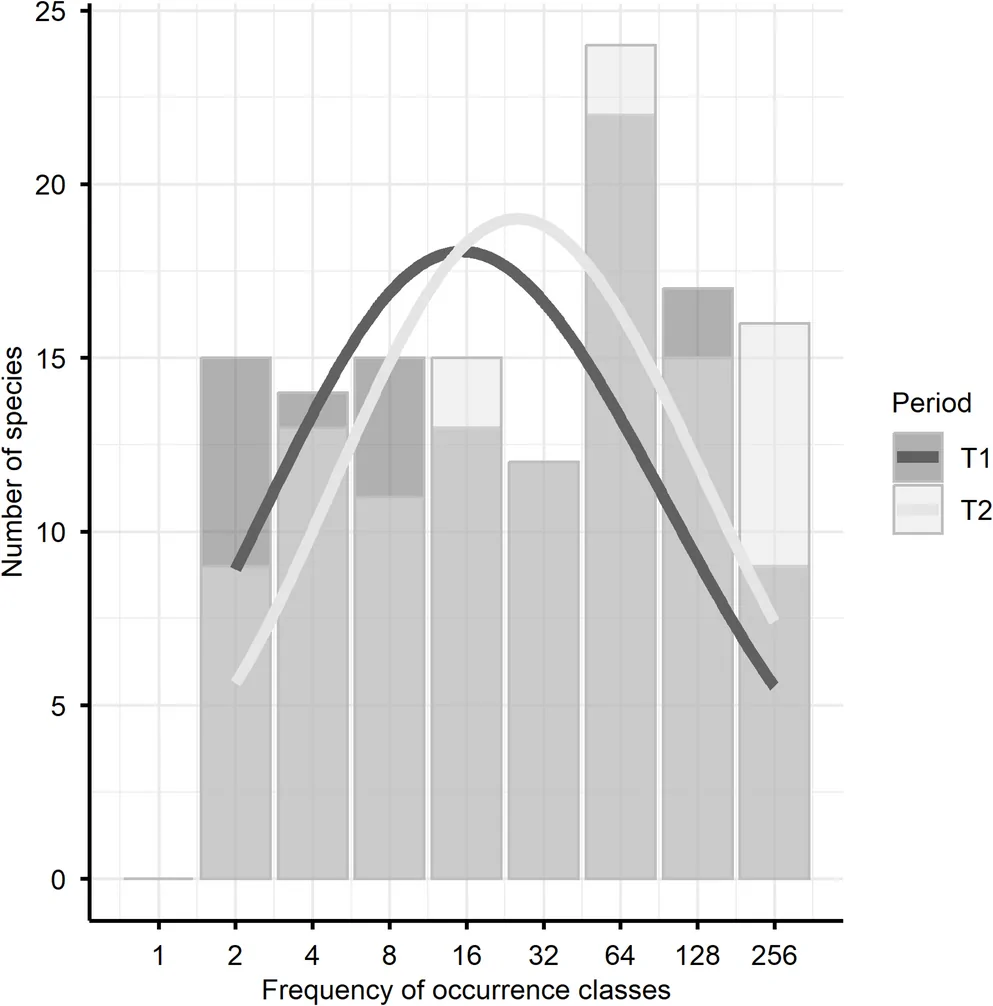
Species Abundance Distribution model (SAD) depicting the distribution of log2 transformed relative abundance (based on frequencies of occurrence classes). Each histogram bin represents a frequency of occurrence class interval on log2 bins (from 0–1 to 128–256). Number of species which fall in each of these intervals is shown in the y-axis. Abundance distributions in histogram bins are juxtaposed for T1 (darker grey) and T2 (lighter grey) with the intermediate grey corresponding to the overlap. Poisson-log-normal model curves for each sampling period were fitted over the histogram (T1, darker grey, T2, lighter grey)

When looking at changes at species level relative abundance, in general there was an increase in species frequency of occurrence (Fig. 3b, “Total” boxplot). More frequent species (Q3 and Q4) showed significant positive net changes compared to the less common groups (Q1 and Q2). This pattern is consistent with the quasibinomial GLM using species relative abundance in T1 as predictor and the relative abundance in T2 as response (Fig. S4, Online Resource 7), in which the probability of a species occurring in T2 increased with the number of sites it occupied in T1 (quasibinomial GLM: slope = 0.032, z = 78, *p* < 0.001). Species abundance distribution models for T1 and T2 further revealed a rightward shift for the T2 model (peak at 16–32 class) in relation to the T1 model mode (peak at 8–16 class), which denoted an increase in the frequencies of occurrence of the typical species in the community (Fig. 4). Species in lower abundance classes (1–2, 2–4 and 4–8) decreased their representativity from T1 to T2, while species in higher abundance classes (32–64 and 128–256) followed the opposite trend.

## Discussion

We found a significant increase in avian alpha diversity in Mediterranean bird assemblages over the past 20 years, with net gains of up to six species per sample unit, representing a ca. 28% rise. These changes restructured avian communities, driven by both the rising prevalence of generalist and forest species, consistent with prior studies (Le Viol et al. 2012; Inger et al. 2015; Gregory et al. 2023; Fig. 3a). This overall pattern reflects a partial recovery through the widespread expansion of adaptable and abundant species (Plieninger et al. 2014; Guilherme and Pereira 2013). Rarefaction analyses further indicated that the main difference between periods lies not in total regional richness, which remained similar between T1 and T2, but rather in the redistribution of species across local sampling units and the increase in local occupancy rates. This increase in alpha diversity related with greater occurrence of common species contrasts with widespread declines across Europe, particularly among farmland specialists and migratory birds, driven by land use intensification, habitat loss and deterioration, and climate change (Brlík et al. 2021; Burns et al. 2021; Rigal et al. 2023).

The observed increase in alpha diversity, together with the rising prevalence of common and adaptable species, may influence community composition by shifting the balance between generalist and specialist taxa, as reported in other Mediterranean landscapes (Santana et al. 2017; Brotons et al. 2018). Generalist species in the study area showed the highest gains in richness and frequency, with similar patterns documented in other depopulated Mediterranean regions consistently promoted generalists while reducing the relative representation of habitat specialists (Davey et al. 2012; Le Viol et al. 2012; Fusco et al. 2021).

Forest birds also increased, including a higher presence of Eurosiberian taxa and species associated with mature forest habitats and improved connectivity, consistent with changes observed in more complex forest habitats and enhanced landscape heterogeneity, and with patterns reported in Mediterranean landscapes (Gil-Tena et al. 2007; Brotons et al. 2018; Burns et al. 2021).

Conversely, farmland birds exhibited a slight overall decline, even though their representation in the sample was low. Nonetheless, some common and fragmentation-tolerant species maintained or even increased their numbers, reflecting a duality between specialist and generalist traits under Mediterranean conditions (Fusco et al. 2021; Oksuz et al. 2021).

Although no direct causal relationships can be inferred from our results, the observed structural shifts in bird communities may align with broader changes in Mediterranean landscapes, namely farming abandonment and passive rewilding (Guilherme and Pereira 2013; Regos et al. 2016; García-Ruiz et al. 2020). The bird community dynamics detected in this study emerged despite a largely stable landscape matrix over the past two decades, with less than 5% variation among forest, shrubland and farmland (Fig.S2, Online Resource 2). A recent remote sensing assessment of the Côa Valley (2006–2022) similarly reported that, despite widespread land abandonment, vegetation change occurred at relatively slow rates and showed strong spatial heterogeneity, with passive regeneration leading to substantial recovery in some areas but remaining limited in others (Elphick et al. 2024).

Recent landscape transformations seem gradual, yet rural landscapes in Mediterranean Europe, including the Côa basin, still carry the legacy of high human presence, intensive land use, recurrent disturbances such as fires, and major rural depopulation driven by sustained migratory exodus, with ecological effects beginning decades earlier (INE 2023; Honrado et al. 2017; Kirkland et al. 2024; FigS.1, Online Resource 1). This legacy delayed the recovery of natural habitats, that although showing a significant increase of generalist and common species, likely constrained the re-establishment of specialist bird communities (Equipa Atlas 2008; Martínez-Abraín et al. 2020).

The last few decades have seen even strong demographic decline and rural outmigration accompanied by reduced labour-intensive agriculture, shifts to low-intensity grazing, increase of open-woodland pastures, fewer wildfires, and less hunting (Torres-Manso et al. 2014; Carboneras et al. 2024; AGIF 2025; EFFIS 2025). This conceals underlying land-use extensification, as rural society moves towards a less managed, more wildlife-compatible landscape (Fernández et al. 2020; Bruno et al. 2021). By enhancing structural heterogeneity and increasing the diversity of available resources, agroforestry systems recently imported from neighbouring Spain, such as the *dehesa/montado* model of larger estates with cattle established there for decades, consistently support higher bird species richness (Santos et al. 2002; INE 2021, 2022; Edo et al. 2023). Similar trends in Mediterranean landscapes link reduced human disturbance with increased avian richness, broader species distributions, a higher prevalence of large-bodied species, and more frequent occurrence of raptors, providing a good example of the patterns observed in our study (Regos et al. 2016; Martínez-Abraín et al. 2020). In the case of the increase of common and generalist species observed here, this pattern may indicate a functional reorganization of bird communities under slowly changing landscape conditions, driven in part by the increasing representation of ecologically flexible and habitat-generalist species, as reported in other Mediterranean landscapes undergoing land abandonment and passive regeneration (Guilherme and Pereira 2013; Elphick et al. 2024).

Though this study is based in a robust sampling design, some limitations should be acknowledged. First, the 1999–2005 baseline was based in sampling data from 13 observers, all experienced ornithologists. In fact, and after raw data was revised, we found high consistency in species lists across observers (Faanes and Bystrak 1981), supporting confident dataset integration. Second, the precise location of original transects is unknown, as GPS was not used in T1. Following national atlas protocols, observers selected routes that maximised habitat diversity within each 2 × 2 km square. In 2023–2024, transect orientations and habitat coverage were replicated as closely as possible. These constraints may add noise but are unlikely to influence the main patterns observed. This interpretation is further supported by the fact that changes were not uniform across the bird community, with contrasting responses among habitat groups and occurrence quartiles between T1 and T2.

## Conclusion

The Côa basin exemplifies rare biodiversity gains in Mediterranean hinterlands, often overlooked due to limited long-term ecological monitoring (Fusco et al. 2021; PECBMS 2025), and often due to difficulties in comparing historical data sets. This study offers the first medium-to-long-term, fine-scale assessment of avian communities in western Iberia’s interior, establishing a critical baseline for future research. Findings reveal a significant increase in local bird species richness over two decades, driven by generalist and forest-associated species. Lower grazing pressure and fewer wildfires have facilitated the expansion of wooded pastures (*montado*) and mitigated human-wildlife conflicts in the Côa basin. In addition, there has been a reduction in hunting pressure over the last few decades. This reflects the “rural paradox” where positive ecological dynamics emerge within seemingly stable land-cover mosaics, masking structural landscape changes driven by rural depopulation and declining agro-pastoral practices (Martínez-Abraín et al. 2020; Bruno et al. 2021). In depopulated territories, wildlife richness and ecosystem services recover following human retreat. These trends position the Côa basin as a model for analogous Mediterranean landscapes, which could serve as refugia for biodiversity-friendly extensification. The region offers opportunities for rewilding, protected-area expansion, and initiatives like the Côa Valley National Park, aligning with EU restoration goals (Jepson et al. 2018; Fernández et al. 2020). However, a critical question remains: do this biodiversity gain signal the recovery of resilient, functionally diverse ecosystems, or merely the rise of adaptable generalists? This uncertainty underscores the urgent need for sustained ecological monitoring to guide future conservation and restoration strategies in rewilding landscapes.

## Supplementary Information

Below is the link to the electronic supplementary material.

Supplementary Material 1: Additional materials supporting this study are organized into seven thematic resources: Online Resource 1 – Socioeconomic and disturbance time series for the Côa River Basin, 1940–2025 (Fig. S1). Online Resource 2 – Land-use transition diagram based on COS 1995–2018 land-use data (Fig. S2). Online Resource 3 – Baseline occurrence data and presence–absence matrices for T1 and T2 (Tables S1–S2). Online Resource 4 – Functional guilds and ecological traits for bird species (Table S3). Online Resource 5 – Species occurrence change categories and ecological classifications (Table S4). Online Resource 6 – Rarefaction and extrapolation curves based on Hill numbers q = 0, q = 1 and q = 2 (Fig. S3).

## Data Availability

All Supplementary Information is archived in the Zenodo Digital Repository https://doi.org/10.5281/zenodo.20159286), where all seven Online Resources (OR1-OR7) are available in their final form. Raw breeding bird atlas data are not included due to national copyright restrictions. These can be formally requested from: Instituto Conservação da Natureza e das Florestas: https://www.icnf.pt.

## Code Availability

Not applicable. All statistical analyses were performed using standard R packages.
